# An Acidic Thermostable Recombinant *Aspergillus nidulans* Endoglucanase Is Active towards Distinct Agriculture Residues

**DOI:** 10.1155/2013/287343

**Published:** 2013-07-10

**Authors:** Eveline Queiroz de Pinho Tavares, Marciano Regis Rubini, Thiago Machado Mello-de-Sousa, Gilvan Caetano Duarte, Fabrícia Paula de Faria, Edivaldo Ximenes Ferreira Filho, Cynthia Maria Kyaw, Ildinete Silva-Pereira, Marcio Jose Poças-Fonseca

**Affiliations:** ^1^Department of Cellular Biology, Institute of Biological Sciences, University of Brasilia, 70.910-900 Brasilia, DF, Brazil; ^2^Department of Biochemistry and Molecular Biology, Institute of Biological Sciences, Federal University of Goias, 74.001-970 Goiania, GO, Brazil; ^3^Department of Genetics and Morphology, Institute of Biological Sciences, University of Brasilia, ET 18/25, Darcy Ribeiro University Campus, 70.910-900 Brasilia, DF, Brazil

## Abstract

*Aspergillus nidulans* is poorly exploited as a source of enzymes for lignocellulosic residues degradation for biotechnological purposes. This work describes the *A. nidulans* Endoglucanase A heterologous expression in *Pichia pastoris*, the purification and biochemical characterization of the recombinant enzyme. Active recombinant endoglucanase A (rEG A) was efficiently secreted as a 35 kDa protein which was purified through a two-step chromatography procedure. The highest enzyme activity was detected at 50°C/pH 4. rEG A retained 100% of activity when incubated at 45 and 55°C for 72 h. Purified rEG A kinetic parameters towards CMC were determined as *K*_*m*_ = 27.5 ± 4.33 mg/mL, *V*_max_ = 1.185 ± 0.11 mmol/min, and 55.8 IU (international units)/mg specific activity. Recombinant *P. pastoris* supernatant presented hydrolytic activity towards lignocellulosic residues such as banana stalk, sugarcane bagasse, soybean residues, and corn straw. These data indicate that rEG A is suitable for plant biomass conversion into products of commercial importance, such as second-generation fuel ethanol.

## 1. Introduction

One of the major challenges of modern society is to promote economic growth in a sustainable model. Global demands of energy consumption stimulate the research on alternative fuels, aiming the reduction of the dependence on nonrenewable energy sources. For some decades now, Brazil and the USA have successfully produced bioethanol from sugarcane and corn, respectively. Nonetheless, plant biomass generated by extensive cultures, and which is not totally converted into useful by-products such as fertilizers and animal feed, tends to accumulate and cause environmental problems. Numerous efforts have been made in order to develop biotechnological routes to produce the so-called second-generation bioethanol from agriculture residues such as corn stover, rice straw, sorghum bagasse, corncobs, wheat bran, wheat straw, and sugarcane bagasse. The limiting step of this process is the availability of low-cost efficient enzymes to convert lignocellulose into fermentable glucose units.

Filamentous fungi can produce and secrete enzymes which efficiently degrade cellulose, a linear polymer of glucopyranose units connected by **β**-1,4 bonds, to oligosaccharides and glucose. Based on model organisms from the genera *Trichoderma *and *Phanerochaete*, fungi cellulolytic enzymes acting in synergism have been classified as (1) endoglucanases or endo-**β**-1,4-glucanases (EC 3.2.1.4), responsible for the random attack of internal glycosidic bonds of the cellulose amorphous region, generating oligosaccharides of various sizes and new chain ends for the action of a second class of enzymes, (2) cellobiohydrolases (EC 3.2.1.91), which processively degrade the reducing and nonreducing ends of amorphous or crystalline cellulose regions, releasing glucose or cellobiose, and (3) **β**-glucosidases, which hydrolyze cellobiose and other small oligosaccharides into glucose. More recently, swollenins, which are proteins homologous to plant expansins, were reported to cooperate in cellulose hydrolysis by fungi such as *T*. *reesei*, *Trichoderma pseudokoningii*, *Trichoderma asperellum,* and *Aspergillus fumigatus*.

Members of the genus *Aspergillus* have been described as efficient cellulases producers. *Aspergillus nidulans* produces three endoglucanases, four cellobiohydrolases and one **β**-glucosidase. Lockington et al. [1] demonstrated that, like in many other cellulolytic fungi, *A*. *nidulans* cellulase genes expression is regulated by the carbon and the nitrogen sources. In a cocultivation study involving the bacterium *Pectobacterium carotovorum*, a proteomic approach revealed that *A*. *nidulans *was the main responsible for leave litter decomposition [2]. Saykhedkar et al. [3] have recently demonstrated that *A*. *nidulans* produces and secretes a complete set of enzymes capable of degrading cellulose, hemicelluloses, and pectin present in sorghum stover, without chemical pretreatment of this substrate. These data point out to *A*. *nidulans *as a candidate for plant biomass conversion at the industrial level.

*A. nidulans* Endoglucanase A (EG A) gene was cloned and characterized: it comprises a 1228 bp sequence interrupted by four introns [4]. The corresponding enzyme presented 35 kDa, displayed the highest activity at 50°C/pH 6.5, and retained 50% of activity when incubated at 30–70°C for 1 h.

Aiming the production of high levels of *A. nidulans* EG A, which could allow a refined biochemical characterization of this potential industrial biocatalyst, in this work we expressed *A. nidulans egl*A cDNA in the *Pichia pastoris* heterologous system. *P. pastoris* has been widely described as a robust and efficient producer of recombinant proteins which are secreted to the culture supernatant. The purified recombinant EG A (rEG A) showed the highest CMCase activity at 50°C and pH 4. It also displayed a remarkable thermostability, retaining almost 100% of activity after 48 h of incubation at the optimum temperature range. Purified rEG A kinetic parameters towards CMC were also determined. Furthermore, we could detect the release of reducing sugars from the incubation of the *P*. *pastoris* recombinant strain crude extract with agricultural wastes such as banana stalk, sugarcane bagasse, soybean residues, and corn straw. These features indicate that *A. nidulans* rEG A is suitable to biotechnological processes such as second-generation biofuel production.

## 2. Materials and Methods

### 2.1. Microorganisms and Growth Conditions

Conidia (10^6^/mL) of the *A. nidulans paba*A1, *bi*A1, *meth*G1, and *arg*B strain were inoculated in Pontecorvo's minimal medium (MM), supplemented with 1.5 g/L hydrolyzed casein, 10 g/L glucose, 2 g/L peptone and 0.5 g/L yeast extract, and incubated at 30°C with agitation for 24 h. Mycelia were then washed with distilled water and inoculated in MM enriched with 1 g/L ball-milled steam-exploded sugarcane bagasse (SCB) for 24 h (30°C/200 rpm) for cellulase genes induction. Sugar cane bagasse was obtained from the Jardinópolis Alcohol and Sugar Mill (JARDEST, São Paulo, Brazil) and was prepared by treatment with superheated steam, followed by instantaneous decompression in a reactor system, as described by Kling et al. (1987) [5]. Processed SCB samples were kept at 4°C.

The *P*.* pastoris* GS115 (*his*4) strain was used as heterologous host according to the conditions described in the *Pichia* Expression kit (Invitrogen, Carlsbad, CA, USA).

For cloning experiments and plasmid manipulations, *Escherichia coli* XL10Gold {TetrD  (*mcrA*)*183*  D(*mcrCB*-*hsdSMR-mrr*)173  endA1 *supE44  thi-1  recA1  gyrA96  relA1  lac*  Hte  *"[" F*′*  proAB  lacI*q*ZDM15*  Tn*10 *(Tet^r^)  Amy  Cam^r^]} (Stratagene, La Jolla, CA, USA) was used. Bacteria were grown at 37°C, in LB medium [5 g/L yeast extract, 10 g/L peptone and 10 g/L NaCl] supplemented with the appropriate antibiotics, when necessary.

### 2.2. Synthesis, Cloning, and Expression of the *A. nidulans egl*A cDNA

After induction for cellulase genes, *A. nidulans *total RNA was extracted using the Trizol reagent (Invitrogen). Synthesis and amplification of the *egl*A cDNA were performed by RT-PCR using the specific primers EGA-*Sna*BI (5′-TACGTAGCTTTCACATGGTTTGG-3′) and EGA-*Avr*II (5′-CCTAGGTTATTGACTTCCCACG-3′), whose design was based on the *egl*A gene sequence described by Chikamatsu et al. [4] (accession no. AB009402). The underlined bases indicate the restriction enzymes recognition sites. A 1.2 kb cDNA molecule was amplified and cloned into the pGEM-T vector (Promega, Madison-WI). After transformation of *E. coli* XL10 Gold competent cells, the *egl*A/pGEM-T plasmid DNA was extracted and digested with *Sna*BI and *Avr*II in order to be transferred to the *P. pastoris* pPIC9 expression vector. In this plasmidial construct, named *egl*A/pPIC9, the *A. nidulans egl*A cDNA was placed under control of the methanol-inducible *AOX*1 promoter in frame with the *Saccharomyces cerevisiaeα*-factor signal peptide encoding sequence (*Pichia *Expression Kit, Carlsbad, CA, USA). After DNA sequencing confirmation, the *egl*A/pPIC9 plasmid was used to transform the *P. pastoris *GS115 strain (Invitrogen, Carlsbad, CA, USA), according to the supplier's recommendations. One hundred transformant clones were grown and screened for efficient EG A-enzyme secretion, as previously described [6]. 

The recombinant clone presenting the highest CMCase activity and one negative control (a *P. pastoris* clone harboring the empty pPIC9 vector) were selected for further analyses.

### 2.3. Purification of the Recombinant EG A from the *P. pastoris* Culture Supernatant

An isolated colony of the *P. pastoris *recombinant strain harboring the *egl*A/pPIC9 construct was grown in 100 mL of BMGY medium [100 mM potassium phosphate pH 5.0, 13.4 g/L YNB (Invitrogen), 0.0004 g/L biotin, 10 mL/L glycerol], in 1-L flasks, incubated at 30°C at 250 rpm until the culture reached an OD_600_ value of 1.0. Cells were then harvested and washed two times with distilled water, resuspended in 100 mL of BMMY medium in 1-L flasks and incubated under the same conditions for additional 48 h, with the addition of methanol to a final concentration of 0.5% (v/v) at every 24 h in order to maintain the induction condition. Finally, cells were centrifuged at 12,000 g/4°C for 15 min, the supernatant was collected, and 0.2 g/L sodium azide was added.

The rEG A purification procedure was based on two systems of ultrafiltration membranes followed by a two-step chromatographic protocol. Initially, the supernatant was applied into an ultrafiltration system employing a membrane with molecular weight cut-off of 50,000 Da (Biomax-50 NMWL, Millipore), under pressure of 2.5 kgf/cm^2^, at 10°C. The MW50 eluted fraction was concentrated on a MW10 ultrafiltration membrane with the molecular weight cut-off of 10,000 Da (Biomax-10 NMWL, Millipore) and then submitted to gel filtration chromatography in a Sephadex G50 column (60.0 × 2.7 cm) equilibrated with 0.5 M sodium phosphate buffer pH 7.0, 25 mM NaCl, at a 20 mL/h flux, at 28°C. The eluted fractions were tested for CMCase activity and protein concentration (A280 nm). Fractions presenting CMCase activity were then pooled, dialyzed (Dialysis tubing D9402, Sigma Aldrich), and applied onto an ionic exchange column (Q-Sepharose 15.0 × 2.5 cm) previously equilibrated with 0.5 M sodium phosphate buffer pH 7.0, at a 20 mL/h flux, at 28°C. The eluted fractions displaying CMCase activity were pooled and employed for the recombinant enzyme biochemical characterization.

### 2.4. rEG A SDS-PAGE and Zymogram Analyses

The SDS-PAGE protocol was performed according to Sambrook and Russel [7] employing 12% (w/v) polyacrylamide gel followed by coomassie blue R250 or silver nitrate staining [8]. In order to detect enzyme activity, a zymogram assay was performed on a 12% (w/v) polyacrylamide gel containing 0.15% (w/v) CMC (carboxymethylcellulose sodium salt low viscosity; Sigma) as previously described [9]. Prior to the zymogram analysis, the samples were precipitated with 10% TCA and washed twice with cold 100% acetone.

### 2.5. rEG A Biochemical Characterization

The CMCase activity, employing CMC as substrate, was determined by the method described by Mandels et al. [10] and modified by Filho et al. [11]. Analyzed samples consisted of the culture medium supernatant (crude extract, CE) and the purified rEG A obtained as described previously. The activity values correspond to the means of three independent experiments, in three technical replicates. The statistical analysis was performed using ANOVA with 5% level of significance and the SPSS for Windows version 17.0 program.

The amount (mg/mL) of reducing sugars produced in each reaction was determined by the DNS method [12] measured by spectrophotometry at A540 nm (Spectramax M2^e^ (Mol. Dev. Corp., Sunnyvale, CA, USA)), using glucose as standard. One unit of enzyme activity was established as the amount of enzyme that released 1 *μ*mol of reducing sugar per minute per mL, expressed as IU/mL.

Enzyme activity was evaluated at temperatures ranging from 30 to 80°C. Optimal pH was established with the following buffers: 50 mM sodium acetate (pH 4.0–pH 6.5), 50 mM sodium phosphate (pH 6.0–pH 7.0), and 50 mM Tris-Cl (pH 6.5–pH 8.0). The determined optima temperature and pH were employed in the subsequent experiments.

The evaluation of the rEG A thermostability was performed by enzyme preincubation at 45°C, 55°C, 70°C, and 80°C for 3, 12, 24, 48, and 72 h.

The effect of metal ions and other chemicals on the endoglucanase activity was assayed by the addition to the reaction system of 18 mM (the HgCl_2_ concentration which causes 50% inhibition of the rEG A CMCase activity) of the following reagents: AlCl_3_, CaCl_2_, ZnSO_4_, NaCl, CoCl_2_, CuCl_2_, KCl, FeCl_3_, EDTA, SDS, beta-mercaptoethanol, and 1,4-dithio-DL-threitol (DTT).

rEG A substrate specificity was performed using CMC, filter paper (Whatman no. 1; 6 cm × 1 cm straps), xylan, microcrystalline cellulose (Avicel, Sigma), and p-nitrophenyl-**β**-D-glucoside (pNPG) as substrates. The final concentration of reducing sugars was determined as described previously. In order to evaluate the rEG A activity towards the substrate 4-methylumbelliferyl-**β**-D-cellobioside (MUC), a qualitative analysis was performed employing UV light to detect the fluorescent digestion product. 

In all experiments, the values for CMCase activity represent the averages of experimental triplicate.

### 2.6. Determination of rEG A Kinetic Parameters

To determine the rEG A Michaelis-Menten kinetic parameters (*K*_*m*_ and *V*_max⁡_), CMC (concentration ranging from 0 to 35 mg/mL) was employed as substrate in a reaction mixture containing 775 *μ*g purified protein in 50 mM sodium acetate pH 4.0 at 50°C for 30 min. The obtained data were analyzed using the program EnzFitter Windows (Biosoft, Cambridge, UK).

### 2.7. Enzyme Activity towards Agricultural Residues

rEG A capacity to hydrolyze lignocellulosic substrates derived from agriculture was assayed in 50 mL flasks containing 2/3 of substrate solution [0.3 mL 1.0 M sodium acetate buffer pH 4.0, 40 mg of the substrate (banana stem, ball-milled steam exploded sugarcane bagasse, soybean cultivation waste or corn stover) and 3.7 mL distilled water] and 1/3 of *P. pastoris* CE (1.25 U/mL FPAse activity) at the proportions of 25, 50, 75, and 100% in the final volume of 6 mL completed with distilled water. Sodium azide (0.2 g/L) was added to avoid contamination by microorganisms. Reaction mixtures were incubated for 24, 48, 72, and 96 h at 50°C/150 rpm. Aliquots of 0.5 mL were periodically collected.

## 3. Results

### 3.1. Cloning of the *A. nidulans egl*A cDNA and Production of the Recombinant Enzyme

The RT-PCR assay using total RNA from *A. nidulans* grown with 10 g/L SCB as the sole carbon source produced a 1.2 kb cDNA fragment, compatible to the size predicted from the splicing of the four putative introns [4]. This cDNA fragment was cloned into the pGEM-T vector and then transferred to the *P. pastoris* pPIC9 expression vector under control of the inducible promoter *AOX*1.

Based on the highest CMCase, a *P. pastoris* recombinant clone was selected for the next experiments. One clone harboring the empty pPIC9 vector was used as negative control.

*P. pastoris* clones were grown under induction conditions, and culture supernatants were evaluated for enzyme activity during a 120 h period. The *P. pastoris *clone containing the *egl*A/pPIC9 construct presented the highest CMCase activity from 24 h of growth; the same activity was maintained throughout the cultivation period. No enzyme activity was detected for the negative control.

### 3.2. Purification of rEG A

**Figure 1:**
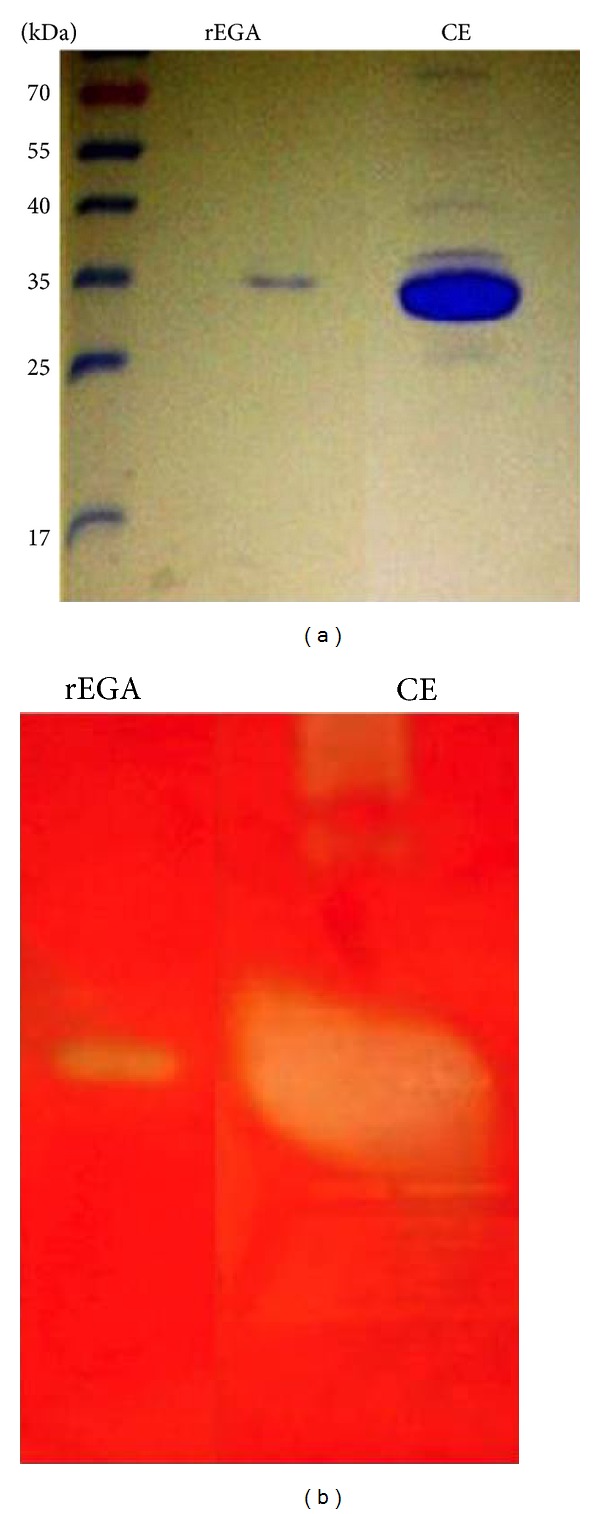
Electrophoretic profile of the recombinant endoglucanase by SDS-PAGE 12% (w/v). The gels were stained with brilliant blue coomassie (a) and activity gel with Congo red (b). MM: molecular mass marker (Fermentas), in kDa; rEG A: sample eluted from Q-Sepharose column; CE: crude extract.

The recombinant *P. pastoris* strain was grown upon induction for 48 h, and the supernatant was applied into a ultrafiltration system employing a membrane with a molecular weight cut-off of 50,000 Da (Biomax-50 NMWL, Millipore) followed by a cut-off membrane of 10,000 Da (Biomax-10 NMWL, Millipore). The obtained sample was named CONCMW10 and was subsequently purified by a two-step separation protocol. After passage through a gel filtration column, an isolated peak of CMCase activity, distinct from the one presenting the highest protein concentration, was obtained (data not shown). Samples corresponding to this activity peak were pooled and submitted to ionic exchange chromatography, which resulted in a sharp peak (data not shown) corresponding to a single protein band of 35 kDa, coincident with the CMC degradation spot in the activity gel (Figure 1).

The four fractions produced by the purification protocol were assayed for CMCase activity and protein concentration. Each fraction specific activities and recovery yield of the recombinant enzyme after the purification steps are summarized in Table 1. After purification, rEG A specific activity was determined as 55.8 IU/mL.

### 3.3. Temperature and pH Effect on rEG A Activity

**Figure 2:**
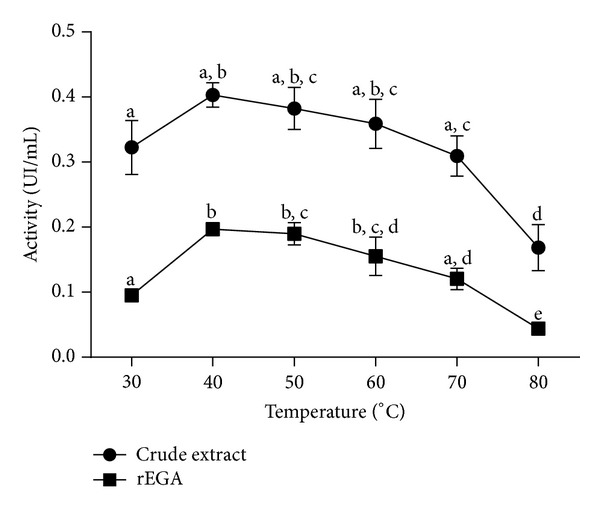
Effect of temperature on the crude extract and rEG A enzyme activity on CMC. The points on the graphs represent the average of experimental triplicates and the vertical bars their standard deviation. The different letters indicate statistical differences between the different assays in the same fraction (*P* < 0.05).

CE and rEG A enzyme activities were analyzed in temperatures ranging from 30 to 80°C, at pH 6.5 (Figure 2). For both samples, the highest CMCase activity was observed when reaction proceeded at 40–60°C. At the extreme temperatures (30 and 80°C), enzyme activity was 50% lower.

**Figure 3:**
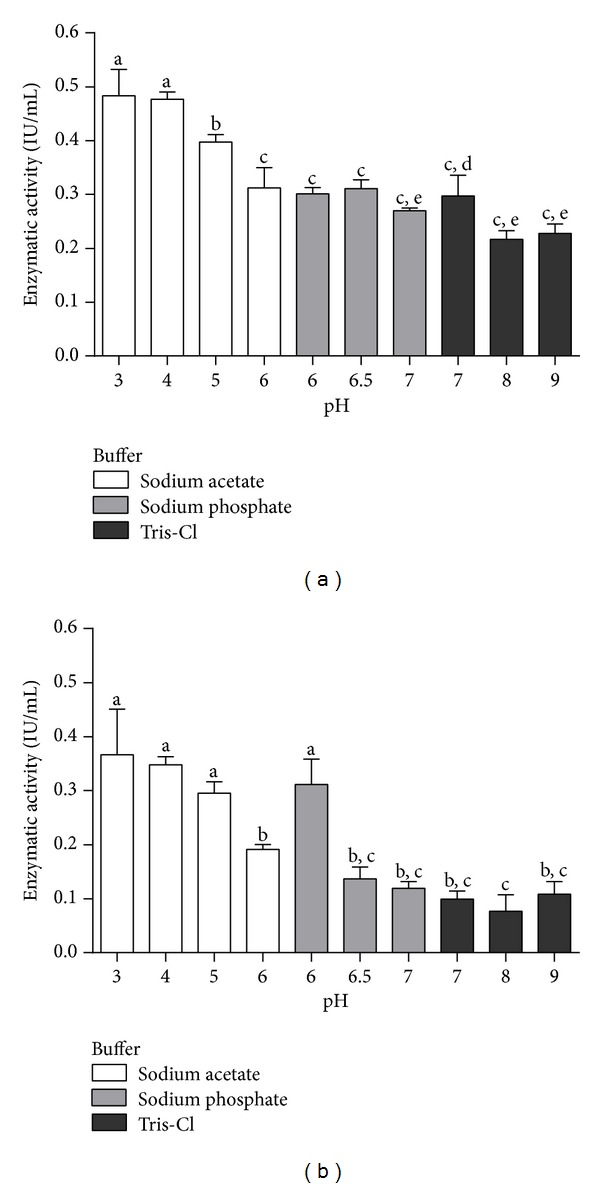
Enzymatic activity of recombinant endoglucanase rEG A (a) and crude extract (b) on CMC. The buffers used were Tris-HCl, sodium acetate or sodium phosphate at the final concentration of 50 mM. The columns represent the averages of experimental triplicate with their corresponding standard deviation. The different letters indicate statistical differences between the different assays (*P* < 0.05).

Enzyme activity towards CMC was assayed from pH 3 to pH 9. Optimum pH was around 4.0 for both enzyme preparations (Figure 3). Alkalinization of the reaction mixture led to a marked decrease in CMCase activity. 

**Figure 4:**
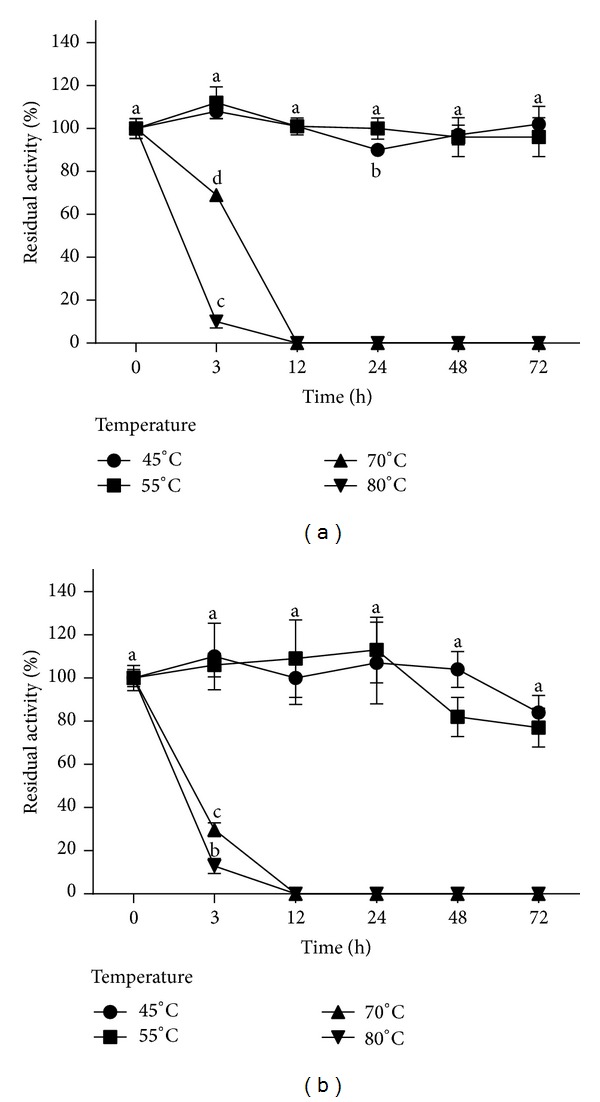
Thermostability of the recombinant endoglucanase, crude extract (a) and rEG A. The results are expressed in terms of residual enzymatic activity (%). The points represent the averages of experimental triplicate with their corresponding standard deviation. The different letters indicate statistical differences between the different assays (*P* < 0.05).

Preincubation of the reaction mixture at 45 and 55°C for up to 72 h did not significantly affect enzyme activity. On the other hand, temperatures of 70°C and 80°C provoked a severe decrease in CMC hydrolysis from the beginning of the preincubation period (Figure 4).

### 3.4. Effect of Metal Ions and Other Chemicals on the rEG A Activity

The effect of cations, chelants, and reducing agents on the purified rEG A activity was assayed (Table 2). All the reagents were tested at 18 mM since this concentration of HgCl_2_ led to a 50% inhibition of the rEG A activity. rEG A CMCase activity was inhibited in 70% by SDS. The reducing agents DTT and beta-mercaptoethanol increased enzyme activity by 32 and 81%, respectively. EDTA did not affect rEG A activity. 

### 3.5. Substrate Specificity of rEG A

**Figure 5:**
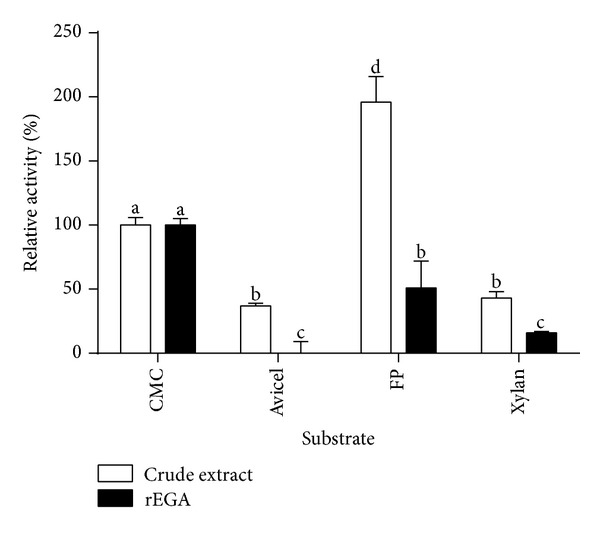
Substrate specific activity of the recombinant endoglucanase. Crude extract and rEG A on carboxymethylcellulose (CMC), microcrystalline cellulose (Avicel), filter paper (FP), and xylan. The different letters indicate statistical differences between the different assays (*P* < 0.05). 100% activity towards CMC as substrate corresponds to 0.28 IU/mL.

Whatman no. 1 filter paper, microcrystalline cellulose (Avicel, Sigma), xylan from oat spelts (Sigma), p-nitrophenyl-beta-D-glucopyranoside (pNPG, Sigma), and 4-methyl-beta-umbelliferyl D-cellobioside (MUC, Sigma) were employed for the rEG A substrate specificity assay (Figure 5). Filter paper activity represented 50% of the activity towards the common endoglucanase substrate CMC, while microcrystalline cellulose and xylan hydrolysis efficiency corresponded to 20% of the verified for this substrate. The recombinant enzyme showed no activity towards pNPG and MUC (data not shown). Recombinant *P*. *pastoris* strain CE presented a significant FPase activity.

### 3.6. rEG A Kinetic Parameters

Increasing CMC concentrations were employed for the determination of the *A*. *nidulans* purified recombinant endoglucanase *K*_*m*_ and *V*_max⁡_ values. With aid of the EnzFitter program, rEG A *K*_*m*_ and *V*_max⁡_ values were determined as 27.5 ± 4.33 mg/mL and 1.185 ± 0.11 mmol/min, respectively.

### 3.7. Enzyme Activity towards Agricultural Residues

**Figure 6:**
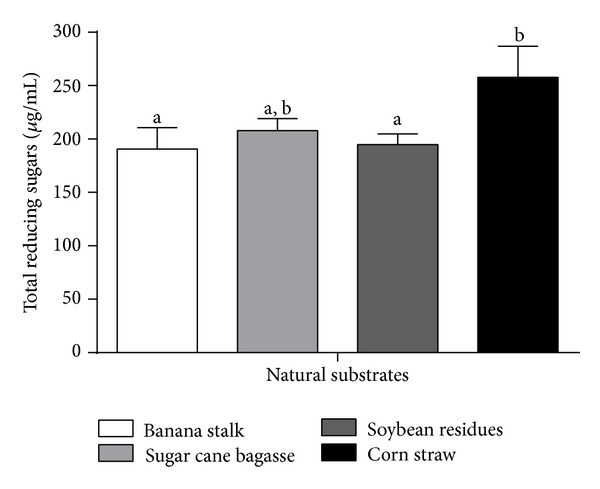
Distinct natural lignocelluloses residues degradation in 72 hours. The experiments were performed in triplicate using the crude extract of *P. pastoris* recombinant. The results are expressed as total sugar formed using the DNS method. The columns represent the averages of experimental triplicate with their corresponding standard deviation. The different letters indicate statistical differences between the different assays (*P* < 0.05).

*P. pastoris* recombinant strain CE was assayed for the capacity of hydrolyzing the natural substrates banana stem, ball-milled steam-exploded sugarcane bagasse, soybean residues, and corn stover. Aliquots from the reaction mixtures were collected after 24, 48, 72, and 96 h of incubation, and released total reducing sugars (TRSs) were quantified. The 72 h incubation period was identified as the most efficient for the release of TRS when the rEG A CE was added at the proportion of 100% (Figure 6). Corn stover was the lignocellulosic substrate more susceptible to enzyme hydrolysis: 250 *μ*g/mL of TRS, a value significantly higher when compared to other agriculture residues. TRSs released from banana stem, sugarcane bagasse, and soybean residues were in the range of 200 *μ*g/mL. In terms of hydrolysis percentage, TRS value corresponded to 3.87% of the corn stover mass present in the assay.

## 4. Discussion

The major impediment for an economically feasible second-generation bioethanol production is the development of strategies to break down the chemical bonds of the polysaccharides that tightly form the cell wall, thus producing free sugars that could be fermented by the already standardized protocols employing *S*.* cerevisiae*. In this view, the formulation of enzyme cocktails which could efficiently degrade cellulose and hemicellulose, the major components of natural residues such as sugarcane bagasse and corn straw, is strategically important. In order to achieve an optimized hydrolysis rate, glycosyl hydrolases prospection and characterization pipelines should be guided by the feedstock composition. Although refined data on sugarcane bagasse composition are not available, sugarcane leaves and culms present about 30% cellulose, 10% pectins, and 50% hemicelluloses [13]. Such a heterogeneous composition, which can vary according to the soil characteristics and cultivation conditions, justifies the prospection for new enzymes presenting distinct biochemical properties, such as peculiar substrate specificities and different optima temperature and pH. In this view, enzyme diversity is of pivotal importance to the design of enzyme cocktails capable of converting sugar cane residues in useful by-products at the industrial level.

In this work, we have cloned the *A. nidulans* endoglucanase A cDNA in *P. pastoris*. The heterologous host was able to produce and to secrete the enzyme in its active form upon induction with 0.5% methanol. rEG A maximum activity was achieved within 24 h of induction, and it was maintained up to 120 h, which is advantageous for several industrial applications. This result also validates the heterologous expression approach since we achieved a large scale of enzyme production in a short period of time. In native secretion systems, or even in heterologous expression models, other studies reported the maximum cellulase activity in the supernatant after longer periods of induction [14–16].

Subsequently, we have performed a two-step ultrafiltration, followed by a two-step chromatography purification procedure. The first ultrafiltration step resulted in a sample (UFMW50) presenting a CMCase specific activity more than seven times higher than the concentrated one (CONCMW50). The same effect was not observed for the second ultrafiltration probably because the enzyme passed through the membrane pore. The ability of CMCase to penetrate an ultrafiltration membrane may be due to its compact structure and/or nonuniformity of membrane pore size.

The chromatographic step consisted in gel filtration which separated two distinct enzymatic peaks (data not shown). In the fractions present in the first peak, specific activity value increased almost 45 times (Table 1). The overall recovery level and fold purification of rEG A were 2.1% and 17.5, respectively. The low yield value was mainly due to the loss of enzyme activity in the ultrafiltration step.

rEG A optimal temperature range (40–60°C, Figure 2) indicates that it corresponds to a mesophilic enzyme [14]. Mesophilic endoglucanases are useful in several biotechnological processes, such as in the formulation of biostoning and biopolishing agents for the textile industry. Furthermore, rEG A maintained 100% of the enzyme activity after a 48 h preincubation period at 45 and 50°C (Figure 4). Thermostability was also described for other recombinant fungal endoglucanases produced in *P*. *pastoris* [16–18], and this characteristic is important for industrial purposes since the enzyme can work efficiently for long periods, without requiring addition of more enzymes to the process.

The optimum pH (4.0) we described for rEG A does not match the value (pH 6.5) observed for the partially purified native EG A [4]. Some endoglucanases from *Aspergilli* display higher activity in the acidic pH range [19, 20]. According to Hahn-Hägerdal et al. [21] and Dashtban et al. [22], acidophilic enzymes are more suitable to industrial lignocellulose degradation, since most of the substrate is pretreated with inorganic acids. 

The reducing agents **β**-mercaptoethanol and DTT provoked 80 and 32% increase in enzyme activity, respectively (Table 2); this suggests that disulfide bonds are not of pivotal importance in rEG A three dimensional structure stabilization. EDTA did not significantly affect rEG A function, possibly indicating that it is not a metalloprotein, as it is the case of* Aspergillus terreus* strains M11 and DSM 826 endoglucanases which are inhibited by this ions chelator [20, 23]. As it was reported for other fungal endoglucanases [24–27], *A*. *nidulans* rEG A interaction with Ca^2+^, Co^2+^ and Cu^2+^ resulted in increased enzyme activity, possibly because these ions exert a stabilizing effect on the enzyme structure without interfering in the catalytic site.

rEG A was able to degrade CMC and, to a lesser extent, filter paper (Figure 5). Some other studies have also reported the activity of fungal endoglucanases towards filter paper [23, 24, 27], which would represent a better substrate for cellobiohydrolases. The ability to hydrolyze different substrates can be explained by nonspecific bindings in the active site and/or by the presence of distinct catalytic domains, each one presenting a particular activity [28]. Our research group has recently demonstrated that the *Humicola grisea* var. *thermoidea* recombinant cellobiohydrolase 1.2 produced in *P*. *pastoris* acts as a bifunctional enzyme, presenting activity towards crystalline and amorphous cellulose [18]. Such dual enzymes can be particularly useful to several bioconversion processes. *A*. *nidulans* rEG A presented no significant activity towards pNPG, xylan, and Avicel. Interestingly, in the recombinant *P*. *pastoris* CE, we detected both FPase and xylanase activities; this is possibly due to unspecific enzyme activities present in the host secretome by itself and/or interaction with the recombinant endoglucanase [29, 30]. Salinas et al. [31] detected a background CMCase activity in the supernatant of a *P*. *pastoris* recombinant strain harboring an empty expression vector; the authors attributed such an activity to *P*. *pastoris* genomic sequences encoding for glycoside hydrolases.

The specific activity we have determined for *A*. *nidulans* rEG A (55.8 IU/mg) is higher than the value observed for another fungal endoglucanase expressed in *P*. *pastoris* for biotechnological purposes, the *Trametes versicolor* recombinant enzyme (35–40 IU/mg) [31]. When compared to industrial enzymes, whose data are normally not available due to patents confidentiality, rEG A specific activity towards CMC was 2 times higher than Spezyme #3 (Genencor Intl) and more than 15 times higher than Biocellulase A (Quest Intl) [32]. These data corroborates rEG A as a potential biocatalyst.

The recombinant *P. pastoris* CE was assayed for the degradation of natural lignocellulosic biomass: banana stem, steam-exploded sugar cane bagasse, soybean residue, and corn stover. The degradation of residues by holocellulases is an efficient and inexpensive process to obtain products with high added value, such as the ones derived from pulp and paper industry, second-generation biofuels, composting, food and feed, among others [33]. However, these residues recalcitrance hampers the access of hydrolytic enzymes in order to release monomeric sugars, especially glucose, to be fermented in subsequent processes [34, 35]. rEG A best degradation efficiency occurred for corn stover. The different hydrolysis efficacy presented for the distinct plant biomass residues possibly reflects the complexity of the lignocellulose composition and its structural arrangement. According to Mansfield et al. [36], the efficacy of enzyme complexes to hydrolyze natural substrates is linked to the innate structural characteristics of the substrate and/or to the modifications that occur during the pretreatment or the saccharification steps.

rEG A activity towards different agriculture residues may also be related to the lignin content of these substrates cell wall. According to Howard et al. [37], lignin represents 15% of rice straw and corn cobs, 30–40% of nut shells, and 25–35% of softwood stems biomass. Lignin is possibly the main responsible for the apparently low percentage of corn stover mass conversion (3.87%, which corresponds to 0,25 g/L) into reducing sugars by rEG A. 

In addition, degradation efficiency by rEG A enzyme could be optimized by different pretreatment schemes or/and the association of other enzymes, such as cellobiohydrolases and xylanases, in the context of enzyme cocktails.

Although native *A*. *nidulans* endoglucanase A had been partially characterized in a previous study [4], most of the features described here for the recombinant enzyme were unknown. Thus, this work provided novel and more complete information about an endoglucanase with biotechnological potential due to the optimum temperature range, the acidic optimum pH, the thermostability, and the capacity to degrade even nonpretreated natural residues such as corn stover.

## Figures and Tables

**Table 1:** Purification of the recombinant endoglucanase from the supernatant of *P. pastoris*.

Fractions	Total protein (*µ*g)	Recombinant endoglucanase A activity			
		Total activity (IU)	Specific activity (IU/mg)	Yield (%)	Purification fold (x)
Crude extract	36.69	116.94	3.2	100	1
Concentrated fraction (MW50)	19.97	9.45	0.5	ND	ND
Ultrafiltered fraction (MW50)	21.35	77.04	3.6	65.9	1.1
Concentrated fraction (MW10)	12.51	3.95	0.3	3.4	0.1
Ultrafiltered fraction (MW10)	24.66	89.57	3.6	ND	ND
Sephadex G50	0.31	4.33	14.1	3.7	4.4
Dialyzed	0.36	4.12	11.5	3.5	3.6
Q-Sepharose	0.04	2.40	55.8	2.1	17.5

ND: not determined.

**Table 2:** Effect of treatment with different agents (chelators, metal ions, detergents, and reducing agents) on rEG A activity.

Treatment	Relative activity (%)
Control	100.00 ± 3.95
SDS	31.24 ± 4.73*
EDTA	103.69 ± 4.07
DTT	132.43 ± 3.11*
FeCl_3_·6H_2_O	92.00 ± 6.09
AlCl_3_	67.10 ± 4.54*
CaCl_2_	112.55 ± 6.91*
ZnSO_4_	83.03 ± 7.19*
NaCl	97.14 ± 4.30
CoCl_2_·7H_2_O	132.26 ± 3.19*
CuCl_2_	120.37 ± 2.33*
KCl	99.34 ± 4.20
*β*-mercaptoethanol	181.81 ± 11.09*

Asterisks (*) indicate statistical difference within the same fraction (*P* < 0.05) when compared to control. The results are presented in terms of activity ± standard deviation. The endoglucanase activity was assayed after the addition of 18 mM of the agents to the reaction system.
